# Neuroprotectant Effects of Hibiscetin in 3-Nitropropionic Acid-Induced Huntington’s Disease via Subsiding Oxidative Stress and Modulating Monoamine Neurotransmitters in Rats Brain

**DOI:** 10.3390/molecules28031402

**Published:** 2023-02-01

**Authors:** Wael A. Mahdi, Shareefa A. AlGhamdi, Amira M. Alghamdi, Syed Sarim Imam, Sultan Alshehri, Mohammad A. Almaniea, Baraa Mohammed Hajjar, Fahad A. Al-Abbasi, Nadeem Sayyed, Imran Kazmi

**Affiliations:** 1 Department of Pharmaceutics, College of Pharmacy, King Saud University, Riyadh 11451, Saudi Arabia; 2 Department of Biochemistry, Faculty of Sciences, King Abdulaziz University, Jeddah 21589, Saudi Arabia; 3 Experimental Biochemistry Unit, King Fahd Medical Research Center, King Abdulaziz University, Jeddah 21589, Saudi Arabia; 4 School of Pharmacy, Glocal University, Saharanpur 247121, India

**Keywords:** 3-nitropropionic acid, hibiscetin, Huntington’s disease, neuroprotection

## Abstract

Background: Previously reported data suggest that hibiscetin, isolated from *roselle*, contains delphinidin-3-sambubioside and cyanidin-3-sambubioside including anthocyanidins and has a broad range of physiological effects. In this study, we aim to analyze the effect of hibiscetin neuroprotective ability in rats against 3-nitropropionic acid (3-NPA)-induced Huntington’s disease (HD). Methods: To investigate possible toxicities in animals, oral acute toxicity studies of hibiscetin were undertaken, and results revealed the safety of hibiscetin in animals with a maximum tolerated dose. Wistar rats were divided into four groups (*n* = 6); (group-1) treated with normal saline, (group-2) hibiscetin (10 mg/kg) only, (group-3) 3-NPA only, and (group-4) 3-NPA +10 mg/kg hibiscetin. The efficacy of hibiscetin 10 mg/kg was studied with the administration of 3-NPA doses for the induction of experimentally induced HD symptoms in rats. The mean body weight (MBW) was recorded at end of the study on day 22 to evaluate any change in mean body weight. Several biochemical parameters were assessed to support oxidative stress (GSH, SOD, CAT, LPO, GR, and GPx), alteration in neurotransmitters (DOPAC, HVA, 5-HIAA, norepinephrine, serotonin, GABA, and dopamine), alterations in BDNF and cleaved caspase (caspase 3) activity. Additionally, inflammatory markers, i.e., tumor necrosis factor alpha (TNF-α), interleukins beta (IL-1β), and myeloperoxidase (MPO) were evaluated. Results: The hibiscetin-treated group exhibits a substantial restoration of MBW than the 3-NPA control group. Furthermore, 3-NPA caused a substantial alteration in biochemical, neurotransmitter monoamines, and neuroinflammatory parameters which were restored successfully by hibiscetin. Conclusion: The current study linked the possible role of hibiscetin by offering neuroprotection in experimental animal models.

## 1. Introduction

Huntington’s disease (HD) is a genetic disorder that causes the progressive breakdown of nerve cells in the brain [1]. HD is a congenital, uncommon, degenerative inherited NDDs illness that can be inherited and lethal. HD is caused by the amplification of the nucleotides, adenine, and guanine (CAG) trinucleotide on chromosomal 4p, which is found in the gene coding (HTT) [2]. Diagnostic evidence shows dyskinesia, cognitive dysfunction, and gradual memory loss. Furthermore, the significantly impacted neurons descend into this cortex from the striatal medium spiny area [3]. Additionally, it is characterized by involuntary movements, cognitive decline, and psychiatric symptoms such as depression and irritability. The individual in the mature phase had great trouble with synchronization, rigidity, little motion, occasional convulsions, confusing articulation, and feeding issues, impairment of judgment skills, confused thinking, and the commencement of psychological conduct [4]. It typically begins in middle age and worsens over time, ultimately leading to severe disability [5]. HD is a neurodegenerative illness that affects memory and sensory deterioration and eventually leads to mortality [6]. Motor, cognitive, and psychological impairments are the most common signs of HD. HD manifests itself in individuals at an early age and eventually leads to death with progressive growth which develops over several years [7]. The trinucleotides were discovered to be replicated 10 to 35 times in the genomes. The individual having HD had a redundancy of genome with multiple repeats. Whenever the doubling becomes little than 40, it became assumed that there were no or few indications of HD, but a larger amount suggested the existence of HD [8]. The extent of the HTT protein enhanced significantly to the replication of the CAG trinucleotide that appears to be aberrant. Additionally, the splitting of the longer protein backbone into tiny particles led to the production of microscopic proteins, hazardous components, that were subsequently implanted in the neuron [9]. As a result, the regular functioning of the neuron is disrupted, which might cause damage and, as a result, the formation of HD [10]. Several neurologic disorders are associated with HD, which results from an upsurge in CAG replications in the huntingtin gene (HTT) [11]. Studies have explored the relationship between a human chromosome (4p16.3) mutation and HD, which is known to cause aberrant N-terminal extension of the CAG triplet that encodes the poly Q tract of the huntingtin protein 1 (HTT1) [12]. Furthermore, evidence indicates that CAG plays a role in HD, with overexpression and a sustained repeat of CAG exceeding 36 leading to the emergence of HD symptoms. While mutant huntingtin may be detected in a variety of brain and peripheral tissues, earlier research has revealed that mHTT accretion and toxicity largely disturb medium GABAergic spiny nerves and, to a slighter extent, cerebral cortex neurons [13]. In HD, there was a significant decrease in GABA transmitter and encephalin neurons within the basal ganglia. The prevalence of HD was higher in North America and the UK than in Asian [14]. The progression of HD commences with the amplification of sequences beyond 50. Exceptionally unregulated behavior was observed in this era [15].

The researchers discovered that patients with HD have dendritic fluctuations in the basal ganglia and spiny neurons with reduced spine density in the brain regions [16,17,18]. Furthermore, brain imaging studies in HD patients and controls suggest that alterations in the cortico-striatal connection may contribute to depression and functional impairments [19,20,21]. The importance of extended poly glutamate stretch associated with neuronal toxicity in HD has also been discussed by several other studies in addition to the involvement of mHTT in the HD pathogenesis [22,23].

In addition to cortical communication, the striatum serves primarily as a relay center for motor and cognitive functions. According to studies, neuronal cell loss in the striatum causes neurodegeneration, which is an important hallmark of HD [24,25]. Previous reports explore that a series loss of synapses is among the primary causes of the onset of HD [26]. Thus, changes in glutamatergic, dopaminergic (DA), and cholinergic synapses may be observed during HD’s pre-symptomatic phase [27,28,29,30,31,32]. Although HD may be detected, there is no proven cure for this condition. Preclinical and clinical research on fetal neural transplantation has been conducted however, the results have been unsatisfactory [33,34].

The mitochondrial toxic compound 3-NPA permanently blocks the succinate dehydrogenase (complex II) enzyme in mitochondria [35]. In earlier data, it is apparent that humans and animals treated with the fungal toxin 3-NPA showed HD-like symptoms. Preclinical investigations using rotarod and locomotor activities revealed that rats administered with 3-NPA showed noteworthy modifications in motor functions. The evidence, therefore, implies that 3-NPA treatment is either effective at reversing early stages or at reversing future phases of HD-linked behavior in humans. The exact mechanism by which 3-NPA causes HD-like symptoms in rats is not fully understood, but it is thought to involve the subsiding of oxidative stress and the modulation of monoamine neurotransmitters in the brain. Oxidative stress is an imbalance between the production of reactive oxygen species (ROS) and the body’s ability to neutralize them, and it is thought to play a role in the development of HD. Additionally, monoamine neurotransmitters, such as dopamine and serotonin, are involved in the regulation of movement, emotion, and other functions, and their levels are often altered in HD [36,37]. Earlier published studies suggested that natural compounds slow the progression of HD by regulating mitochondrial activity, reducing peroxidation, and activating the immune [38]. Many researchers asserted that systemic administration of 3-NPA mimics major pathological aspects of HD, particularly mitochondrial dysfunction, oxidative stress, and specific striatal atrophy [35]. As a result, it is the benchmark for experimental induction of HD in rats.

Medicinal herbs and natural remedies have played an essential part in daily living for ages. About 25–30% of prescription medications are derived from plant-based sources, which serve a significant role in the mitigation of various forms of diseases [39]. Because of their nutritional qualities and bioactive chemical sources [39], comestible flowers have been documented as unique dietary components, primarily anthocyanin, nitrogenous chemicals, and carotenoids [40]. Flavonoids as a low molecular weight phytoconstituent proven to be an fundamental amount of human dietary nutrition [41]. Flavonoids have also been found to have antioxidant and chelating activities, which may offer protection from life diseases [42]. The Malvaceae family member *Hibiscus rosa sinensis* is a medicinal as well as an ornamental plant with an array of clinical benefits. Many phytochemical constituents of Hibiscus have been isolated and characterized. Flavonoids, alkaloids, saponins, tannins, and polyphenols are among those found in Hibiscus [43]. In addition, roselle anthocyanin has health benefits with its antioxidant and bioactive properties [44,45]. Hibiscetin, which is isolated from Roselle (*Hibiscus sabdariffa*), contains delphinidin-3-sambubioside and cyanidin-3-sambubioside [39], both of which are known as natural food colorants and important purification components, as per previous findings [46]. The spectral studies established the structural component of hibiscetin-3-glucoside (C21H20O14) as an important flavonoid compound that is derived from the petals of *Hibiscus rosa sinensis* and exhibits potent anticancer activities [47]. According to a recent study, hibiscetin can reduce the severity of Parkinson’s disease in rats induced-rotenone paradigm [48]. The current orthodox treatment available for the HD is inadequate and expensive, with some identified demerits, hence there is need to identify the potential component from natural origin to deal with severe neurodegeneration that led in to clinical conditions like HD. The current study was to investigate the effects hibiscetin against 3-NPA-induced HD in rodents by evaluating the biochemical alterations, i.e., glutathione (GSH), superoxide dismutase (SOD), catalase activity (CAT), lipid peroxidation (LPO), GSSG reductase (GR), and inflammatory markers, i.e., tumor necrosis factor alpha (TNF-α), interleukins beta (IL-1β), and myeloperoxidase (MPO).

## 2. Results

### 2.1. Acute Toxicity Assessment

In the study, hibiscetin was found to remain safe in acute toxicity studies in rats. During the 14-day acute toxicity phase, neither morbidity nor clinical manifestations were seen. We selected 10 mg/kg of hibiscetin for the the main investigation based on results of the acute oral toxicity research.

### 2.2. Mean Body Weight

**Figure 1:**
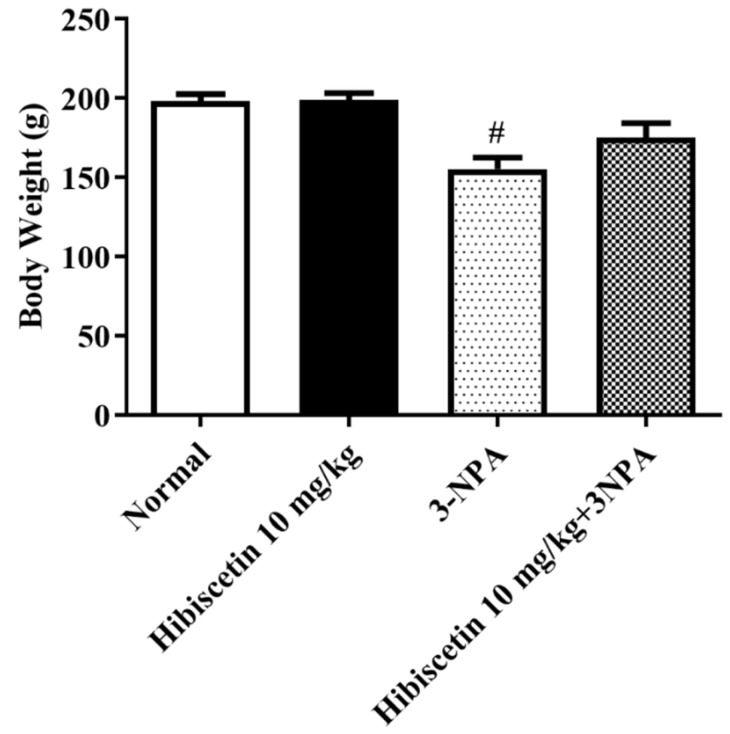
The impact of hibiscetin on mean body weight in rats with Huntington’s disease-like symptoms caused by 3-nitropropionic acid. Values are expressed as mean ± S.E.M. (*n* = 6). Values are statistically non-significant at *p* > 0.05 vs. negative control group # *p* < 0.05, respectively (one-way ANOVA followed by Tukey’s test).

A remarkable reduction in rat body weight was seen in the rats in the 3-NPA control group as hibiscetin was applied to rats to induce Huntington’s-like characteristics (Figure 1). Rats treated with hibiscetin (10 mg/kg) showed no significant changes observed in the weight as compared to 3NPA treated rats. 

### 2.3. Oxidative Stress Parameters

**Figure 2:**
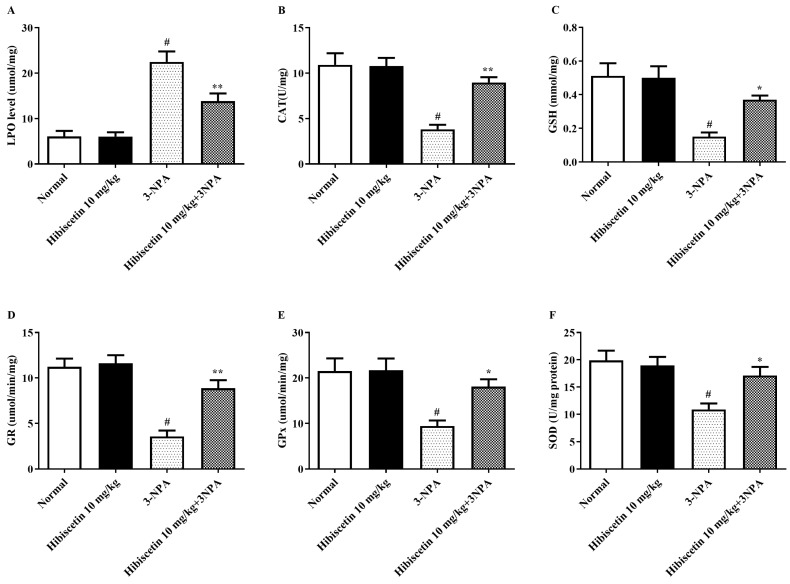
The impact of hibiscetin on lipid peroxidation and antioxidant markers in rats with Huntington’s disease-like symptoms caused by 3-nitropropionic acid (**A**) LPO, (**B**) CAT, (**C**) GSH, (**D**) GR, (**E**) GPx, (**F**) SOD. Values are expressed as mean ± S.E.M. (*n* = 6). Values are statistically significant at * *p* < 0.05, ** *p* < 0.01 vs. negative control group # *p* < 0.05, respectively (one-way ANOVA followed by Tukey’s test).

Figure 2A–F depicts an assessment of the impact of hibiscetin on oxidative disequilibrium measures in animals with HD-like symptoms produced by 3-NPA. While compared with the control group, 3-NPA-induced rodents displayed a significant elevation in (*p* < 0.05) enzymatic activity, with a focus on peroxidation activity as a key indication of free radical formation. Additionally, other set groups showed significant declines in oxidative biomarkers in brain tissue of 3-NPA-induced rats, including CAT, GSH, GR, GPx, and SOD (*p* < 0.05). In addition to that, one-ANOVA and post hoc assessment shown that timely hibiscetin (10 mg/kg) given for 15 days lead to a substantial drop in LPO (*p* < 0.01) and remarkable restoration in the CAT (*p* < 0.01), GSH (*p* < 0.05), GR (*p* < 0.01), GPx (*p* < 0.05), and SOD (*p* < 0.05) when equated against the 3-NPA group. Hibiscetin (10 mg/kg) per se shows no substantial effects.

### 2.4. Monoamine Metabolites and Caspase 3 Biomarkers Estimation

**Figure 3:**
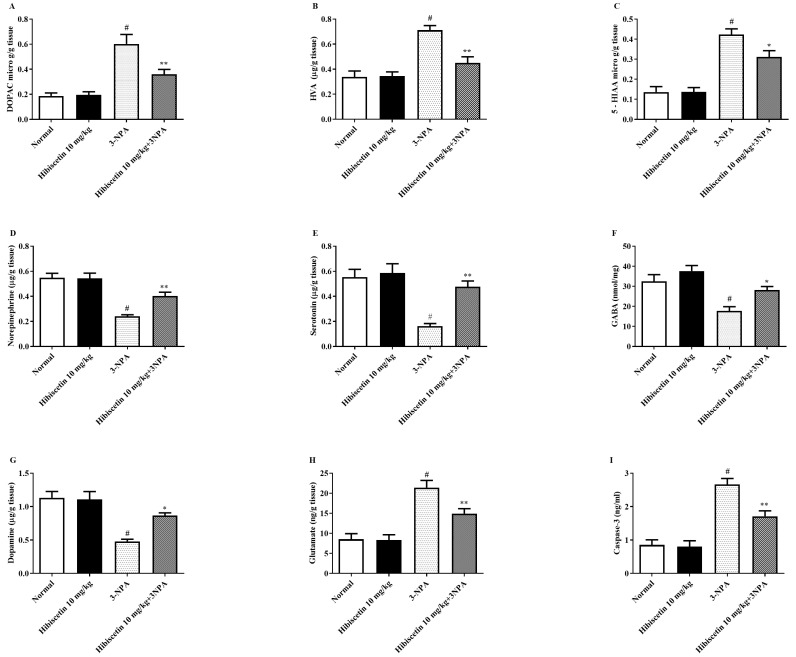
The impact of hibiscetin on monoamines and free amino acid content in rats with Huntington’s disease-like symptoms caused by 3-nitropropionic acid (**A**) DOPAC, (**B**) HVA, (**C**) 5-HIAA, (**D**) norepinephrine, (**E**) serotonin, (**F**) GABA, (**G**) dopamine (**H**) glutamate (**I**) caspase 3. Values are expressed as mean ± S.E.M. (*n* = 6). Values are statistically significant at * *p* < 0.05, ** *p* < 0.01 vs. negative control group # *p* < 0.05, respectively (two-way ANOVA followed by Tukey’s test).

Figure 3A–I depicts the effect of hibiscetin on neurotransmitter and amine contents in animals with HD-like symptoms caused by 3-NPA. In the current investigation, we found that when compared to control rats, 3-NPA-induced rats exhibited increased levels of specific monoamines such as DOPAC, HVA, 5-HIAA, and glutamate underlining the importance of 3-NPA in neurological changes. Additionally, 3-NPA-induced rats exhibits a considerable decline in the several monoamine levels including norepinephrine, serotonin, GABA, and dopamine as a hallmark for the 3-NPA-induced neurotoxicity. One-way ANOVA and parametric test (Tukey’s) explore that regular hibiscetin (10 mg/kg) therapy was significantly restored DOPAC (*p* < 0.01), HVA (*p* < 0.01), 5-HIAA (*p* < 0.05) and glutamate (*p* < 0.01) intensifies when compared to the 3-NPA group. When comparison to a 3-NPA group, there was noteworthy modification in norepinephrine (*p* < 0.01), serotonin (*p* < 0.01), GABA (*p* < 0.05), and dopamine (*p* < 0.05).

Figure 3 h depicts the caspase 3 activity displaying HD-like symptoms, as well as the probable effects of hibiscetin on these levels. In the current investigation, we revealed that as compared to control rats, 3-NPA-induced animals had a significant rise in the cleaved caspase 3 antibody activity highlighting the role of 3-NPA. Furthermore, the one-way ANOVA and parametric measures findings indicate that routine hibiscetin (10 mg/kg) intervention leads to a remarkable modulation in the caspase 3 activity when matched with the 3-NPA group. Hibiscetin (10 mg/kg) per se shows no significant effects.

### 2.5. Estimation of Inflammatory Biomarkers 

**Figure 4:**
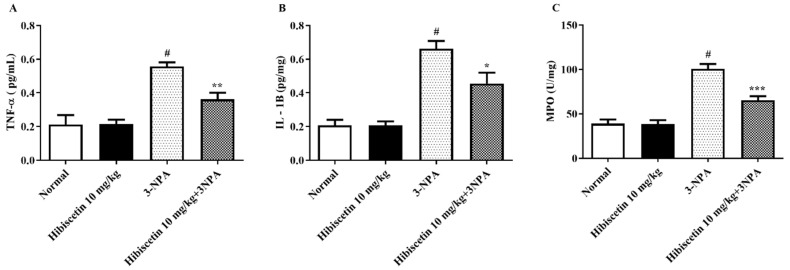
The impact of hibiscetin on inflammatory parameters in rats with Huntington’s disease-like symptoms caused by 3-nitropropionic acid (**A**) TNF-α, (**B**) IL-1β, (**C**) MPO. Values are expressed as mean ± S.E.M. (*n* = 6). Values are statistically significant * *p* < 0.05, ** *p* < 0.01, *** *p* < 0.001 vs. negative control group # *p* < 0.05, respectively (one-way ANOVA followed by Tukey’s test).

Figure 4A–C depicts the measurement of proinflammatory factors in rats with HD-like symptoms generated by 3-NPA, in addition to the possible activities of hibiscetin in moderating the relevant degrees. In the current study, we discovered that 3-NPA-induced animals exhibited substantially greater values of several proinflammatory cytokines including TNF-α, IL-1β, and MPO than control rats, demonstrating the involvement of 3-NPA in proinflammatory markers. Furthermore, one-way ANOVA and post hoc analysis demonstrated that routine hibiscetin (10 mg/kg) shows results in a significant decline in TNF-α (*p* < 0.01), IL-1β (*p* < 0.05), and MPO (*p* < 0.001), as compared to the 3-NPA control group. Hibiscetin (10 mg/kg) alone did not produce significance.

### 2.6. Estimation of BDNF Activity 

**Figure 5:**
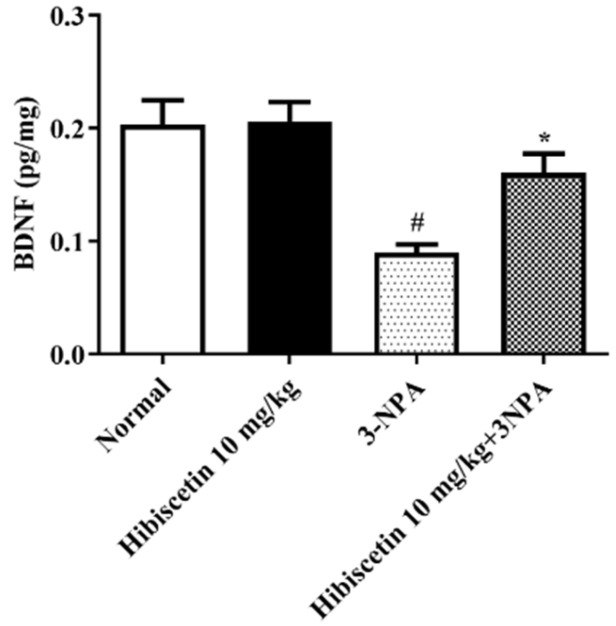
The impact of hibiscetin on BDNF activity in rats with Huntington’s disease-like symptoms caused by 3-nitropropionic acid values are expressed as mean ± S.E.M. (*n* = 6). Values are statistically significant at * *p* < 0.05, vs. negative control group # *p* < 0.05, respectively (one-way ANOVA followed by Tukey’s test).

Figure 5 portrays BDNF activity in rats having HD triggered by 3-NPA, as well as the potential effects of hibiscetin on the aforementioned levels. When compared to control mice, 3-NPA-induced rodents demonstrated a significant reduction in BDNF expression. We found that the use of hibiscetin (10 mg/kg) treatment resulted in a significant modulation of BDNF activity in rats compared to 3-NPA control rats according to one-way ANOVA and post hoc parametric test (*p* < 0.05). Hibiscetin (10 mg/kg) alone did not produce significance.

## 3. Discussion

Researchers looked at hibiscetin as an impending neuroshielding agent for 3-NPA-induced HD in an experimental paradigm. As part of the screening process, hibiscetin toxicity tests were conducted to test for potential hazards related to the substance; no adverse reactions were observed. An earlier study has highlighted the neurotoxic potential of 3-NPA, a mitochondrial hazardous toxin known to trigger HD manifestations. A rodent study with 3-NPA showed that it strongly produced symptoms similar to HD. Preclinical studies suggested that the administration of 3-NPA profoundly affected rodent motor capabilities. Our study showed substantial modifications in 3-NPA-treated rats, such as a noticeable decrease in mean body weight compared to normal control rats. Data show that a change, i.e., a drop in body weight, is an essential feature for the HD [49]. 

A number of biochemical evaluations are included in the current study in order to shed light on the shifts that are linked to HD through experimental models. Several biochemical factors that contribute to HD pathogenesis are outlined in the testimony. The biochemical parameters mainly emphasize the evaluation of oxidative stress, assessment of monoamines metabolites, and estimation of inflammatory markers. Earlier studies explored the involvement of several oxidative parameters as a substantial hallmark in the pathology of HD, with great emphasis on GSH, SOD, CAT, LPO, GR, and GPx in the brain and neuronal tissues. Further, studies also underlined that being an important hallmark of HD there is a substantial decline in the levels of several biomarkers such as CAT, GSH, GR, and SOD in 3-NPA-induced experimental animal models for HD [50,51]. The current study found a substantial decrease in CAT, GSH, GR, GPx, and SOD activity in the rat brain in 3-NPA-treated animals, as well as higher levels of LPO. We suggested that hibiscetin may be protective efficacy in HD-like symptoms following post-treatment with hibiscetin for 15 days after induction of 3-NPA. Similarly, previous results revealed that a change in neurotransmitter levels occurred as an observable event during chemical-induced neurotoxicity. Furthermore, with special emphasis on monoamines such as serotonin, norepinephrine, dopamine, GABA, and glutamate, which have been identified as important targets in the pathology of HD [52,53,54,55]. A study was conducted to investigate how much monoamines and free amino acids were present in brain homogenate in this study. The injection of 3-NPA dramatically changed the levels of neurotransmitters such as DOPAC, HVA, 5-HIAA, norepinephrine, serotonin, GABA, and dopamine. In contrast, hibiscetin treatment significantly restored the above measures in rats.

BDNF is a protein that plays an important role in the growth, survival, and maintenance of neurons in the brain [56]. In HD, the levels of BDNF are known to be decreased, which contributes to the degeneration of nerve cells and the symptoms of the disease [57]. The reduction of BDNF levels in animals treated with 3-NP is thought to mimic the changes that occur in HD and provides a useful model to study the mechanisms of the disease and to develop new treatments [58]. Earlier research revealed that BDNF levels have dropped in the HD animal model [59]. The drop is attributed to an upsurge in ROS, a reduction in BDNF, and a change in Huntingtin protein activity [60]. Likewise, we found that animals treated with 3-NPA remarkably exhibit the remarkable elevation of caspase activities, which are enzymes that play a role in programmed cell death (apoptosis). This is thought to mimic the abnormal accumulation of toxic proteins that occurs in HD and leads to the death of nerve cells in the brain. Animal paradigm 3-NP-induced HD has helped researchers to understand the underlying mechanisms of the disease and to develop new treatments. Further, pre-treatment with hibiscetin significantly overcomes the above downregulations of elevated levels of caspase 3 activity and restored the BDNF suggestive of its neuroprotective role in 3-NPA-induced neurotoxicity in rats.

Neurodegeneration has been widely associated with neuroinflammation, as there is widely disseminated evidence. In a number of studies, it has been demonstrated that neurodegeneration is associated with marked changes in numerous pro-inflammatory biomarkers, namely TNF-α, IL-1β, and MPO [61,62]. The activation of these pathways is thought to contribute to the degeneration of nerve cells and the development of symptoms in the 3-NP-induced model of HD. Furthermore, the studies have demonstrated that the activation of microglia, the resident immune cells in the brain, and the subsequent release of pro-inflammatory mediators are involved in the pathogenesis of HD. The activation of microglia can lead to the release of toxic substances such as reactive oxygen species and nitric oxide, which can cause damage to nerve cells [63]. The present study demonstrated that hibiscetin regulates the suppressing neuroinflammatory pathway, and maintains the monoamines and BDNF as well as caspase 3 activity in the brain. We exposed that injection of 3-NPA substantially exaggerated the levels of all the biomarkers, which were effectively suppressed by the administration of hibiscetin suggesting this might produce its anti-neuroinflammatory effect. Future molecular levels including Western blots and immunochemistry studies are required to confirm this mechanism. Limitations of the study include the short duration and the small number of animals used.

## 4. Methodology

### 4.1. Animals

Wistar rats male (180 ± 20 g), *n* = 6, were chosen and maintained in an in-house laboratory under standard conditions (25 ± 2 °C, humidity 55–60%). During the current research, rats stayed fed with a standard food pellet meal and were given unlimited supply of water. The recognized Trans-Genica Animal Ethics Commission has authorized rodent research (IAEC/TRS/PT/22/10).

### 4.2. Chemicals

In the existing study, 3-NPA, glutathione-reduced lactate, trichloroacetic acid, and dehydrogenase estimation kits were obtained from Sigma (St. Louis, MO, USA). High-grade hibiscetin (98.0% purity) was used in the study and obtained from SK Lab, Maharashtra, India. Other reagents and chemical compounds from Modern Lab, Maharashtra, India. 

### 4.3. Acute Toxicity Studies

Hibiscetin was assessed for acute oral toxicity using OECD ANNEX-423 standards (LD50). According to recently reported, we used 10 mg/kg of hibiscetin and delivered it orally to rats [64].

### 4.4. Experimental Design

The projected investigation is grounded on formerly conducted studies with slight reforms. Before start of study, the rats from all groups were subjected to a seven-day acclimatization period under controlled laboratory conditions. To induce prompt toxicity in rodents, 3-NPA in saline (pH 7.4) will be administered once daily for 15 days based on previously conducted protocols [65,66,67]. In the current investigation, animals were allocated to four groups (*n* = 6). Among the four groups, (group 1) was treated with normal saline, (group 2) hibiscetin (10 mg/kg) only, (group 3) 3-NPA only, and (group 4) 3-NPA and 10 mg/kg hibiscetin. 

From day 8 to 22 normal treated group received saline 3 mL/kg, 3-NPA control group received 3 mL/kg oral 0.5% sodium CMC post 1h treatment 3-NPA 10 mg/kg., i.p. The test group 10 mg/kg p.o. dose were received hibiscetin in 0.5% SCMC for 15 days. Each day post 1 h of above oral treatments, 3-NPA was injected 10 mg/kg of (i.p.) to groups III and IV. During the protocol, behavioral testing was performed for rats. On the last day, i.e., on day 22, after body weight assessment brains were used for biological assessments.

### 4.5. Body Weights

Rats were weighed at the start of study and at end of the study, i.e., on the 22nd day of the protocol before a biochemical estimation on the last day of the protocol.

### 4.6. Biological Assessment 

#### 4.6.1. Brain Tissue Homogenate

Using ice-cold isotonic saline, the brains of the animals were separated and cleaned. A phosphate buffer (0.1 M, pH 7.3) was cast-off to homogenize isolated brain samples. The sections were centrifuged, and the biochemical evaluation was carried out with the supernatant [68,69].

#### 4.6.2. Brain Biochemical Parameters

The obtained homogenate was subjected to the estimation of various parameters based on provided standard measurement kits including GSH [70], SOD [71], CAT [72], LPO [73], GSSG reductase (GR) [74], and inflammatory markers i.e., TNF-α, IL-1β, and MPO. In addition to the booklets included with the commercially available kits, previous studies describe the estimating technique in detail.

#### 4.6.3. Estimation of Brain-Derived Neurotrophic Factor (BDNF) Activity

As recommended by the manufacturer, ELISA kits designed for rats were cast-off to measure BDNF in the brain tissue homogenate.

#### 4.6.4. Estimation of GPx Activity

Glutathione peroxidase (GPx) action in brain cells was examined using a pre-existing technique [75]. According to the protocol, supernatants from brain tissues were combined with phosphate buffer (75 mM; pH 7.0), GSH (150 mM), glutathione reductase (340 U/mL), EDTA (25 mM), NADPH (5 mM), 20% Triton X-100, and sodium citrate (7.5 mM). A 340 nm measurement was taken of the oxidation of NADPH (extinction coefficient = 6.22 L/mmol/cm) to NADP+ over 3 min. 

#### 4.6.5. Assessment of Monoamines and Caspase 3 Biomarkers 

Several monoamines including Dopamine (DA), GABA, glutamate, norepinephrine (NE), dihydroxy phenylacetic acid (DOPAC), serotonin (5-HT), homovanillic acid (HVA), and 5-hydroxy indole acetic acid (5-HIAA) were estimated using analytical kits (Sigma-Aldrich). The quantity of separate monoamine in μg/gram of brain tissue was determined [76]. The caspase 3 was validated using an ELISA kit and caspase 3 levels biomarkers were measured in nanograms per milliliter.

### 4.7. Statistical Analysis

To examine the data in the present study, we used GraphPad Prism 8.0. The outcomes were measured as mean ± standard error mean (SEM). To find the degree of implication, and to elucidate the differences among the variables of each group, we implemented statistical interpretation via one-way analysis of variance (ANOVA) followed by Tukey’s post hoc test. Statistical implication was well-defined as *p* values less than 0.05.

## 5. Conclusions

Evidence claiming hibiscetin to be a strong neuroprotectant and the capacity to alleviate many biochemical indicators of oxidative damage, inflammation, the altered neurotransmitter in 3-NPA-induced Huntington’s disease, and its related neurotoxicity in experimental mice were assessed for the first time. Interestingly, hibiscetin may be protected against Huntington’s disease-like symptoms in this model via regulating monoaminergic neurotransmission.

This might lead to the development of cost-effective flavonoid options for Huntington’s disease management. Hibiscetin may be effective for treating neurodegenerative diseases such as Huntington’s disease.

## Data Availability

Not applicable.
